# Esculetin Neutralises Cytotoxicity of* t*-BHP but Not of H_2_O_2_ on Human Leukaemia NB4 Cells

**DOI:** 10.1155/2017/9491045

**Published:** 2017-03-07

**Authors:** Virginia Rubio, Ana I. García-Pérez, M. Cristina Tejedor, Angel Herráez, José C. Diez

**Affiliations:** Unidad de Bioquímica y Biología Molecular, Departamento de Biología de Sistemas, Universidad de Alcalá, Alcalá de Henares, 28871 Madrid, Spain

## Abstract

The coumarin esculetin shows antioxidant action on some cell types, both by scavenging ROS and by decreasing ROS production. We have previously demonstrated the induction of apoptosis by esculetin on NB4 human leukaemia cells by an ill-defined mechanism related to ROS levels. In this work, we analyze the effect of the simultaneous treatment with esculetin and two oxidants to observe the early events in the mechanism of esculetin-induced apoptosis. Our results show that, from the early time of 15 min, esculetin acts synergistically with H_2_O_2_ to decrease cell viability and metabolic activity and to increase apoptosis in NB4 cells. In contrast, the early oxidative effects of* t*-BHP are neutralised by esculetin, protecting human leukaemia NB4 cells from apoptosis. Esculetin seems to restrict the increase in peroxides caused by H_2_O_2_ or* t*-BHP in the time interval analyzed. These results contribute to a better understanding of the cytotoxic effect caused by esculetin on NB4 cells. At the same time, the early neutralisation of exogenous oxidants could be of interest to prevent diseases related to oxidative stress imbalance.

## 1. Introduction

Intrinsic and microenvironmental oxidative stress has a critical role in cancer development. Reactive oxygen species (ROS) may support survival of tumour cells but also may kill them. The therapeutic use of the cytotoxic action of ROS is exemplified by therapies based on oxidative stress, such as radiotherapy and photodynamic therapy [1]. The imbalance between the production of ROS and antioxidant defence systems in the cells seems to be also implicated in the pathogenesis of a variety of other diseases like cardiovascular diseases, Alzheimer's disease, Parkinson's disease, human aging, and progression of cancer [2–4]. ROS are also molecules modulating many cellular signalling pathways [1]. Disturbance of intracellular redox balance by both oxidants and antioxidants appears to affect cell fate [5]. Therefore, both pro- and antioxidant therapies have been proposed for treating cancer [6].

It has long been proven that cancer cells display a prooxidative state caused by an enhanced aerobic metabolism [7]. Human NB4 cell line, derived from acute promyelocytic leukaemia, possesses increased ROS levels compared to noncancerous cells [8, 9]. In this cell line ROS promote cellular proliferation by protecting from apoptosis and contribute to cancer development [9]. Thus, a good strategy against this leukaemia might be the use of antioxidant compounds to reduce oxidative stress and promote cell death [6]. A similar approach has been the use of phenolic compounds with antioxidant activity to decrease cell proliferation, by inducing apoptosis, in human erythromegakaryocytic leukaemia cells, characterised by very high intracellular ROS levels [10].

Coumarins are natural benzopyrones that bind weakly to a wide variety of enzymes and receptors in the organism, so exhibiting therapeutic potential as anticoagulant, anticancer, antioxidant, anti-inflammatory, antibacterial, and antiviral activities, among others [11, 12]. Their antioxidant properties with the ability to inhibit lipid peroxidation and to act as ROS scavengers [13, 14] seem to be related to the position and number of hydroxyl groups. Thus esculetin (6,7-dihydroxycoumarin) shows higher antioxidant activity than other coumarins [14]. Esculetin is present in many plants traditionally used in natural medicine, displaying cytoprotective properties against oxidative stress-induced cell damage [14–16], whereas it induces apoptosis in other cell lines such as adipocyte 3T3-L1 cells [17], human leukaemia U937 cells [18, 19], and HL-60 cells [20]. Esculetin shows other biological activities such as inhibition of lipoxygenase and tyrosinase [21, 22].

In a previous study, we demonstrated a proapoptotic activity of esculetin on NB4 cells that produced significant cell death by apoptosis, involving the mitochondrial pathway and modulating ROS [23]. We also studied whether changes in ROS levels could be related to esculetin-induced apoptosis. Pretreatment of NB4 cells with esculetin prevented the oxidative effect of exogenous ROS generated from* tert*-butyl hydroperoxide (*t*-BHP). On the contrary, esculetin potentiated superoxide anion production from H_2_O_2_ as well as apoptosis induced by H_2_O_2_ on NB4 cells. Both oxidants,* t*-BHP and H_2_O_2_, increased ROS in NB4 cells via different mechanisms.* t*-BHP is a short chain analogue of lipid hydroperoxides, similar to peroxidised fatty acids whereas H_2_O_2_ is also generated under oxidative stress conditions modulating fundamental intracellular processes [13]. Therefore, the objective of the current study was to analyze the effect of esculetin acting simultaneously with such oxidants and to observe the early events in the mechanism of esculetin-induced apoptosis. This approach may yield a better understanding of the mechanism of cytotoxicity exerted by esculetin on NB4 cells.

## 2. Material and Methods

### 2.1. Reagents

Esculetin (6,7-dihydroxycoumarin, 98% purity) was obtained from Sigma-Aldrich (Steinheim, Germany), prepared as 196 mM stock solution in dimethyl sulfoxide and stored at −20°C. H_2_O_2_ (hydrogen peroxide) and* t*-BHP (*tert*-butyl hydroperoxide) were also purchased from Sigma-Aldrich and prepared as 880 mM and 78 mM stock solutions, respectively, in distilled water at the time of use. Fluorescent probes hydroethidine (DHE) and 2′,7′-dichlorodihydrofluorescein diacetate (DCFDA) were obtained from Molecular Probes (Eugene, Oregon, USA).

### 2.2. Cell Culture

The NB4 leukaemic cell line was obtained from the American Type Culture Collection (Manassas, VA, USA). Cells were maintained in culture at a density of 3 × 10^5^ cells/ml in RPMI medium (Gibco-Life Technologies) supplemented with 10% foetal bovine serum (FBS), 1% penicillin/streptomycin, and 0.02 mg/ml gentamicin at 37°C in a humidified 5% CO_2_ atmosphere.

### 2.3. Cell Treatments for Flow Cytometric and MTT Analyses

NB4 cells (5 × 10^5^/ml) were treated with 100 *μ*M esculetin and either 1 mM H_2_O_2_ or 100, 250, or 500 *μ*M* t*-BHP. These treatments were carried out independently or in combination, for different times (15 min, 30 min, 1 h, and 2 h). Appropriate controls were run in parallel.

### 2.4. Cell Viability

Viability of NB4 cells treated with esculetin was determined by measuring the level of impermeability to propidium iodide (PI, Calbiochem) by flow cytometry. Fluorescence increase will be due to necrotic cells which are permeable to PI. After the different treatments, 2.5 × 10^5^ cells were washed with 500 *μ*l phosphate buffered saline (PBS) and resuspended in 300 *μ*l PBS; then 15 *μ*l of propidium iodide was added and the fluorescence was measured using a Becton Dickinson FACScalibur flow cytometer (San José, CA, USA) and processed using the WinMDI 2.8 software.

### 2.5. Cell Metabolic Activity

Cell metabolic activity was determined by the colorimetric MTT assay kit (Roche Diagnostics, SL) in which tetrazolium salts (MTT) are reduced to formazan by the mitochondrial succinate-reductase system. Cells were seeded in 96-well microplates and after treatment were incubated with 10 *μ*l of MTT labelling reagent for 4 h, and then the formazan dye formed was dissolved with 100 *μ*l of solubilisation solution. The absorbance was measured using a multiwell spectrophotometer, to estimate the number of viable cells.

### 2.6. Analysis of Apoptosis by Annexin-FITC Cytometry Assay

Apoptosis in NB4 cells was quantified by the presence of phosphatidylserine on the outer side of the membrane using the Annexin V-FITC Apoptosis Detection Kit (BioVision). After treatments with esculetin, 2.5 × 10^5^ cells were sedimented at 1200 rpm for 5 minutes and incubated with 500 *μ*l 1x annexin Binding Buffer and 1 *μ*l annexin-FITC for 5 min at room temperature in the dark. Then, 10 *μ*l of PI was added and apoptotic cells were quantified by fluorescence by flow cytometry as indicated before. The results were analyzed using the WinMDI 2.8 software.

Lower left quadrant (FITC^−^/PI^−^) corresponds to living cells, lower right quadrant (FITC^+^/PI^−^) corresponds to early apoptotic cells, upper right quadrant (FITC^+^/PI^+^) corresponds to late apoptotic cells, and upper left quadrant (FITC^−^/PI^+^) corresponds to necrotic cells. Quadrants were defined based on the major population of live control cells (low PI; low annexin), and their position kept constant for all other samples.

### 2.7. Measurement of Intracellular ROS Levels

To measure intracellular superoxide anion (^•^O_2_^−^), cells were incubated with 2 *μ*M DHE (dihydroethidine) during the last 15 min of the different treatments and fluorescence detected by flow cytometry. Intracellular ROS levels (mainly peroxides) were detected using the fluorescent probe DCFDA (2′,7′-dichlorodihydrofluorescein diacetate), a fluorogenic dye that measures hydroxyl, peroxyl, and other reactive oxygen species (ROS) activity within the cell. After diffusion into the cell, DCFDA is deacetylated by cellular esterases and later oxidised by ROS into 2′,7′-dichlorofluorescein, a highly fluorescent compound. After treatment, cells were incubated with 10 *μ*M DCFDA for 30 min at 37°C and then washed with PBS and fluorescence intensity was measured by flow cytometry.

### 2.8. Statistical Analysis

Data are expressed as the mean ± standard error of the results of at least three independent experiments. Significance of differences was determined using the Tukey-Kramer test. Comparisons between treated and untreated cells are labelled with *∗* in the figures. Comparisons of cells treated with esculetin plus oxidant versus esculetin alone are labelled with +. Comparisons of cells treated with esculetin plus oxidant versus the same oxidant alone are labelled with #. In all cases, one and two symbols indicate *p* < 0.05 and *p* < 0.01, respectively.

## 3. Results

### 3.1. Viability of NB4 Cells Treated with Esculetin, H_2_O_2_, and* t*-BHP

Previous studies showed that the lowest concentration of esculetin that decreased both cell viability (to 75%) and metabolic activity (to 65%) of human leukaemia NB4 cells was 100 *μ*M applied for 19 h [23]. Such a concentration was hence chosen to study the early influence of ROS on the esculetin-induced apoptosis of NB4 cells. To this purpose, cells were treated with esculetin and either H_2_O_2_ or* t*-BHP, alone or in combination with esculetin, for different time intervals ranging from 15 min to 2 h.

**Figure 1:**
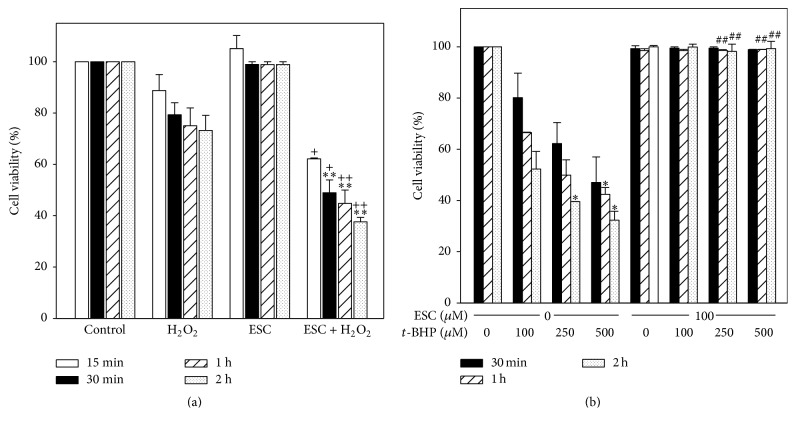
*Cell viability, measured by flow cytometry as impermeability to PI, of NB4 cells treated with esculetin alone or combined with oxidants*. (a) Viability of NB4 cells treated with esculetin alone or combined with H_2_O_2_. Cells (5 × 10^5^/ml) were treated with 100 *µ*M esculetin alone or in combination with 1 mM H_2_O_2_ for different times. (b) Viability of NB4 cells treated with esculetin alone or combined with* t*-BHP. Cells (5 × 10^5^/ml) were treated with 100 *μ*M esculetin, 100, 250, and 500 *µ*M* t*-BHP, or both compounds, for the indicated times. Data are expressed as mean ± SEM of three independent experiments. ^*∗*^Treated versus untreated cells; ^*∗*^*p* < 0.05; ^*∗∗*^*p* < 0.01. ^+^Treated with esculetin plus oxidant versus esculetin alone; ^+^*p* < 0.05; ^++^*p* < 0.01. ^#^Treated with esculetin plus oxidant versus oxidant alone; ^##^*p* < 0.01.

As it can be observed in Figure 1(a), 1 mM H_2_O_2_ produced a decrease in cell viability from 15 min to 2 h of treatment. In this interval, esculetin did not produce any changes in cell viability. However, when cells were simultaneously treated with both H_2_O_2_ and esculetin a significant decrease of viability was observed.

Correspondingly,* t*-BHP (100, 250, or 500 *μ*M) reduced cell viability from 30 min to 2 hours, and such reduction was abolished by esculetin (Figure 1(b)).

Therefore, esculetin is potentiating the effect of H_2_O_2_ while it protects cells from* t*-BHP.

### 3.2. Metabolic Activity of NB4 Cells Treated with Esculetin, H_2_O_2_, and* t*-BHP

**Figure 2:**
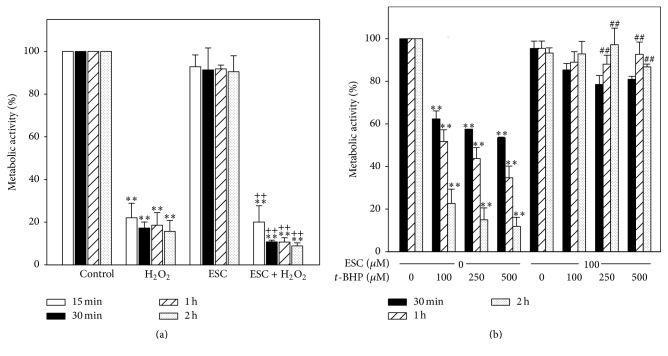
*Metabolic activity, measured by MTT assay, of NB4 cells treated with esculetin alone or combined with oxidants*. (a) Metabolic activity of NB4 cells treated with esculetin and H_2_O_2_. Cells (5 × 10^5^/ml) were exposed to 100 *μ*M esculetin, 1 mM H_2_O_2_, or both, for different times. (b) Metabolic activity of NB4 cells treated with esculetin and* t*-BHP. Cells (5 × 10^5^/ml) were exposed to 100 *μ*M esculetin or 100, 250, and 500 *µ*M* t*-BHP separately or in combination, for the indicated times. Data are expressed as mean ± SEM of four independent experiments. ^*∗*^Treated versus untreated cells; ^*∗∗*^*p* < 0.01. ^+^Treated with esculetin plus oxidant versus esculetin alone; ^++^*p* < 0.01. ^#^Treated with esculetin plus oxidant versus oxidant alone; ^##^*p* < 0.01.

Metabolic activity was measured by the MTT assay. Treatment of NB4 cells with 1 mM H_2_O_2_ reduced dramatically the metabolic activity (Figure 2(a)). Esculetin reduced cell metabolic activity only marginally, even after 2 hours (Figure 2(a)). The simultaneous cotreatment with these two compounds showed a reduction similar to that produced by H_2_O_2_ alone.

Treatment with* t*-BHP also reduced significantly metabolic activity in a concentration- and time-dependent manner (Figure 2(b)). Again, the effect of esculetin alone was weak even at 2 hours. However, when applied simultaneously with* t*-BHP an early moderate reduction of metabolic activity was observed, somewhat reverted a long time.

Hence, esculetin does not prevent the loss of metabolic activity caused by H_2_O_2_ but it does protect cells from* t*-BHP.

### 3.3. Induction of Apoptosis on NB4 Cells Treated with Esculetin and H_2_O_2_

**Figure 3:**
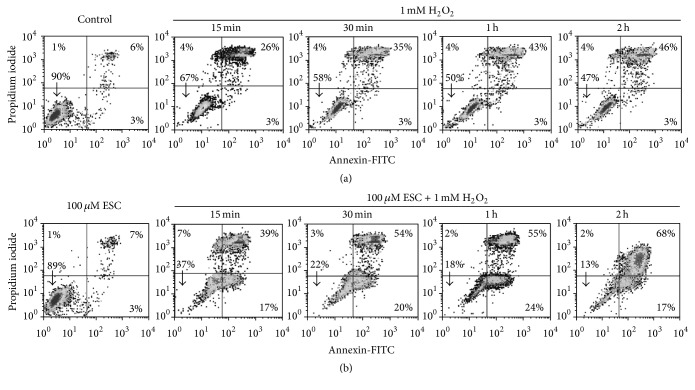
*Apoptosis and necrosis of NB4 cells treated with esculetin and H*_*2*_ *O*_*2*_. Cells (5 × 10^5^/ml) were treated with 100 *μ*M esculetin, 1 mM H_2_O_2_, or the combination of both, for the indicated times. Cells were then stained with FITC-conjugated annexin and PI and measured by flow cytometry. The results show one representative experiment out of three. Vertical axes: propidium iodide intensity (log scale); horizontal axes: annexin-FITC intensity (log scale).

Figure 3 shows that 1 mM H_2_O_2_ produced a time-dependent increase in apoptosis as measured by double labelling with propidium iodide and annexin-FITC (Figure 3(a)). H_2_O_2_ treatment increased apoptosis already at 15 min. Simultaneous treatment with esculetin strengthened apoptosis induced by H_2_O_2_ (Figure 3(b)), mainly early apoptosis. Therefore, esculetin potentiates apoptosis induced by H_2_O_2_ in a time-dependent way.

### 3.4. ROS Production in NB4 Cells Treated with Esculetin and H_2_O_2_

**Figure 4:**
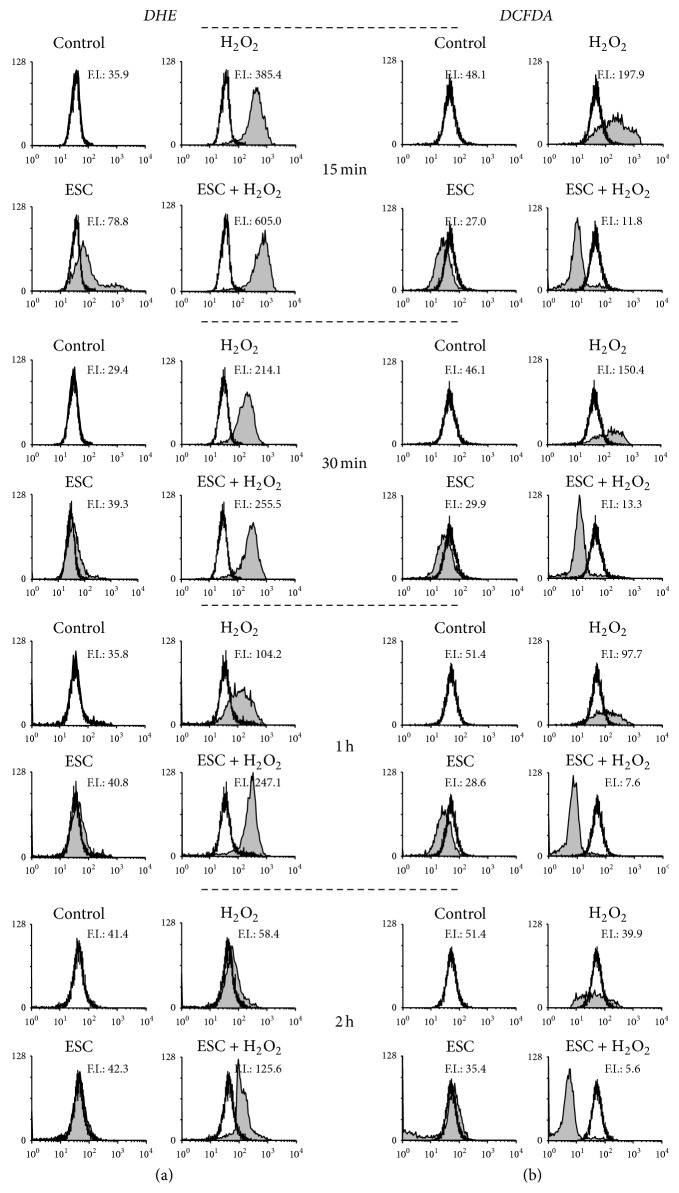
*Intracellular ROS levels in NB4 cells treated with esculetin and H*_*2*_ *O*_*2*_. Cells (5 × 10^5^/ml) were treated with 100 *μ*M esculetin, 1 mM H_2_O_2_, or the combination of both, for different times, and then analyzed by flow cytometry using a specific probe. (a) To measure intracellular superoxide levels, 2 *μ*M DHE was added during the last 15 min of the esculetin treatment. (b) To analyze intracellular peroxides, 10 *μ*M DCFDA was added for 30 minutes at 37°C after the treatments. One representative experiment is shown out of five. Vertical axes: number of events (linear scale); horizontal axes: fluorescence intensity (log scale). F.I. represents mean fluorescence intensity.

Levels of superoxide anion and peroxides were measured using the probes DHE and DCFDA, respectively.  Figure 4(a) shows superoxide anion levels after treatment with esculetin and/or H_2_O_2_. As it can be observed, H_2_O_2_ produced an early and definite increase in superoxide between 15 min and 1 hour. Esculetin did not change the fluorescence profile obtained with DHE in comparison to control cells. Simultaneous cotreatments with esculetin and H_2_O_2_ seem not to have any effects over that produced by H_2_O_2_ alone at any time of treatment.

When using DCFDA as a probe for testing peroxide levels, an early increase is seen at 15 min that later decreases at 2 hours. Esculetin alone does not produce peroxides but in combined treatments with H_2_O_2_ it decreases peroxide levels in comparison to the control cells (Figure 4(b)).

Therefore, esculetin does not neutralise superoxide anions produced by H_2_O_2_ in the time interval analyzed, while it prevents the increase of peroxides.

### 3.5. Induction of Apoptosis on NB4 Cells Treated with Esculetin and* t*-BHP

**Figure 5:**
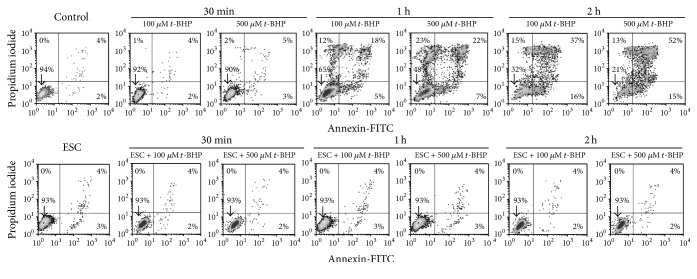
*Apoptosis and necrosis of NB4 cells treated with esculetin and t-BHP*. Cells (5 × 10^5^/ml) were treated with 100 *μ*M esculetin or 100 or 500 *μ*M* t*-BHP separately or in combination, for different times. Cells were then stained with FITC-conjugated annexin and PI and measured by flow cytometry. One representative experiment is shown out of three. Vertical axes: propidium iodide intensity (log scale); horizontal axes: annexin-FITC intensity (log scale).

Figure 5 shows apoptosis induction by treatments with* t*-BHP. As it can be observed,* t*-BHP at both 100 and 500 *µ*M produced a time-dependent and concentration-dependent increase in apoptosis of NB4 cells. However, simultaneous treatment with esculetin did not display apoptosis even for the highest time and concentration of* t*-BHP. Therefore, esculetin is protecting cells against* t*-BHP-induced apoptosis.

### 3.6. Free Radical Production in NB4 Cells Treated with Esculetin and* t*-BHP

**Figure 6:**
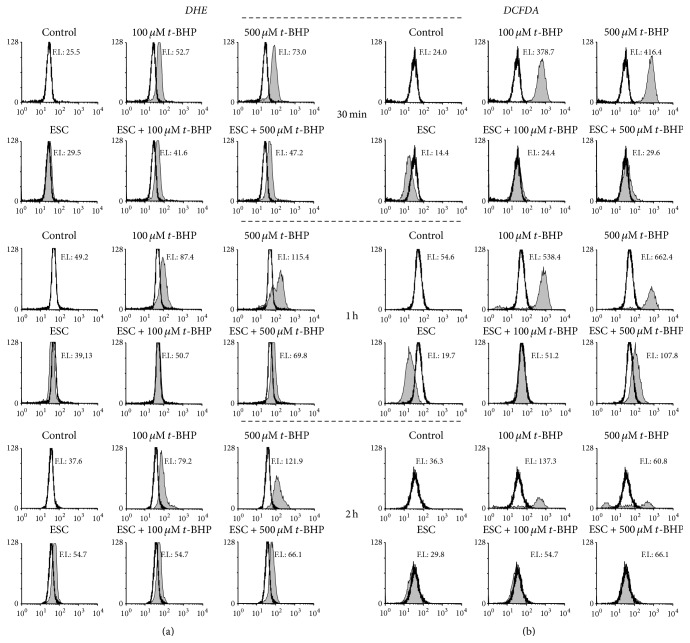
*Intracellular ROS levels in NB4 cells treated with esculetin and t-BHP*. Cells (5 × 10^5^/ml) were treated with 100 *µ*M esculetin or 100 or 500 *µ*M* t*-BHP or the combination of both, for the indicated times, and then analyzed by flow cytometry using a specific probe. (a) To measure intracellular superoxide levels, 2 *μ*M DHE was added during the last 15 min of the esculetin treatment. (b) To quantify intracellular peroxides, 10 *μ*M DCFDA was added for 30 minutes at 37°C after the treatments. One representative experiment is shown out of three. Vertical axes: number of events (linear scale); horizontal axes: fluorescence intensity (log scale). F.I. represents mean fluorescence intensity.

Levels of superoxide anion and peroxides after incubation with esculetin and* t*-BHP were measured (Figure 6). Treatments with* t*-BHP increased both superoxide radical and peroxides in a concentration- and time-dependent way. Simultaneous treatment of NB4 cells with* t*-BHP and esculetin neutralised both superoxide anion and peroxides at any* t*-BHP concentration and time assayed. Esculetin seems hence to compensate the oxidative action of* t*-BHP by neutralising the peroxides generated from it.

## 4. Discussion

We had previously demonstrated that esculetin is able to prevent ROS production in NB4 human leukaemia cells. The reduction of ROS was temporally followed by cell growth arrest and caspase-dependent apoptosis via the intrinsic mitochondrial pathway. Apoptosis was preceded by an early (6 h) loss of mitochondrial membrane potential with a release of cytochrome c to the cytosol [23]. Esculetin also reduced Bcl2/Bax ratio, in correlation with the increased apoptosis. To know whether the antioxidant activity of esculetin can modulate death of NB4 cells, we increased the oxidative stress by adding* t*-BHP or H_2_O_2_ to cells that were pretreated with esculetin. Thus, we have studied the early events (from 30 min to 2 h) of the cotreatment with esculetin and these two exogenous oxidants in order to understand the cytotoxic mechanism of esculetin.

Our results show that, from the earliest time of 15 min of cotreatment, esculetin acts synergistically with H_2_O_2_ decreasing cell viability and metabolic activity (Figures 1(a) and 2(a)) and increasing apoptosis (Figure 3). This suggests a possible implication of ROS in esculetin-induced apoptosis of NB4 cells. H_2_O_2_ increased superoxide anion and peroxides at early times of treatment. That increase of peroxides, but not of superoxide, is neutralised by esculetin, potentiating apoptosis. The increase may be due to production of superoxide anion in the presence of esculetin (Figure 3) in comparison to H_2_O_2_-independent treatment. On the contrary, in human hepatoma HepG2 cells esculetin has been shown to suppress cell damage induced by H_2_O_2_ [24].

It is well known that H_2_O_2_, like some of the most common chemotherapeutic agents, induces ROS generation and cytotoxicity in cancer cells by producing DNA breaks, lipid peroxidation, PARP activation, glutathione depletion, alteration of the energy metabolism, and so on [11, 25]. However, the dose of H_2_O_2_ determines the type of cell death and while low doses trigger apoptosis, higher doses cause severe oxidative modifications which can be irreversible and lead to necrosis [26]. For example, in HeLa cells a low H_2_O_2_ concentration induces caspase-dependent cell death [24].

Although the redox potentials of H_2_O_2_ and, particularly,* t*-BHP are hard to estimate accurately, peroxy radicals are commonly described as weakly oxidising, with a reduction potential ca. 0.43 V or 0.71 V for* t*-BHP^•^ [27, 28]. Hydrogen peroxide is, in contrast, a stronger oxidant (1.78 V for the H_2_O_2_/H_2_O pair). This may account for the different ability of esculetin to neutralise the production of ROS and superoxide from both agents. In addition, there is wide evidence that the actions of both oxidants towards cell damage are exerted in different manner, and H_2_O_2_ affects a wider range of mechanisms, resulting in higher toxicity in some cases [29].

Our results indicate that esculetin potentiates apoptosis of NB4 cells in H_2_O_2_ presence, correlating it with an increase in superoxide anion between 15 min and 1 h (Figure 4). It could be suggested that superoxide increase and therefore oxidative stress are an early event in the apoptotic mechanism of esculetin. On the other hand,* t*-BHP slightly increased the levels of superoxide anion along the 2 h of treatment and peroxides from 30 min to 2 h of treatment. These increases were neutralised by esculetin from the earlier time of 15 min at both low and high* t*-BHP concentrations (Figure 6). Esculetin also neutralised the apoptosis (Figure 5) caused by* t*-BHP. This behaviour differs from that observed for H_2_O_2_.

*tert-*Butyl hydroperoxide is a short chain analogue of lipid hydroperoxides that forms a stable radical species, and it has been widely used to induce oxidative stress in a variety of cells. In hepatocytes* t*-BHP is metabolised by cytochrome P450 generating free radical intermediates such as alkoxyl or peroxyl radicals and superoxide anion [30].* t*-BHP is also metabolised by glutathione peroxidase, leading to glutathione depletion [31]. In erythrocytes,* t*-BHP reacts with haemoglobin forming* t*-butoxyl radical which causes membrane lipid peroxidation and haemolysis [32]. In these and other cells,* t*-BHP also produces alteration of calcium homeostasis and DNA damage [33] leading to cell death via apoptosis or necrosis [16].

Esculetin seems to neutralise these radical species. According to our results, esculetin suppresses cell damage induced by* t*-BHP, as also reported in rat liver [30] and DNA damage by 1,2-dimethylhydrazine in rat colon [16]. The protective effect of esculetin against oxidation by* t*-BHP could be related to prevention of early membrane lipid peroxidation and the intracellular oxidation.

## 5. Conclusions

Combined treatments with esculetin and one of the exogenous oxidants H_2_O_2_ or* t*-BHP show that esculetin neutralises the early oxidative effects of* t*-BHP, protecting human leukaemia NB4 cells from apoptosis, whereas esculetin potentiates the oxidative stress and cell death induced by H_2_O_2_. This suggests a different cytotoxic mechanism for these two oxidants. Any other mechanism independent of ROS scavenging could also be involved in the cytotoxic H_2_O_2_ effects potentiation by esculetin. These results could be relevant to design therapies against diseases related to oxidative stress imbalance.

## Abbreviations

Annexin-FITC: Fluorescein isothiocyanate coupled to annexin V
DCFDA: 2′,7′-Dichlorodihydrofluorescein diacetate
DHE: Hydroethidine
PI: Propidium iodide
MTT: 3-(4,5-Dimethylthiazol-2-yl)-2,5-diphenyltetrazolium bromide
ROS: Reactive oxygen species
*t*-BHP: * tert*-Butyl hydroperoxide.
